# Supplementary material for: Genomic alterations and evolution of cell clusters in metastatic invasive micropapillary carcinoma of the breast
Source: Nat Commun. 2022 Jan 10;13:111. doi: 10.1038/s41467-021-27794-4 (PMC8748639; doi:10.1038/s41467-021-27794-4)
Supplement: Supplementary file 1 — Supplementary Information [file 41467_2021_27794_MOESM1_ESM.pdf]

SUPPLEMENTARY INFORMATION

Genomic Alterations and Evolution of Cell Clusters in Metastatic Invasive Micropapillary Carcinoma of the Breast

Qianqian Shi et al.

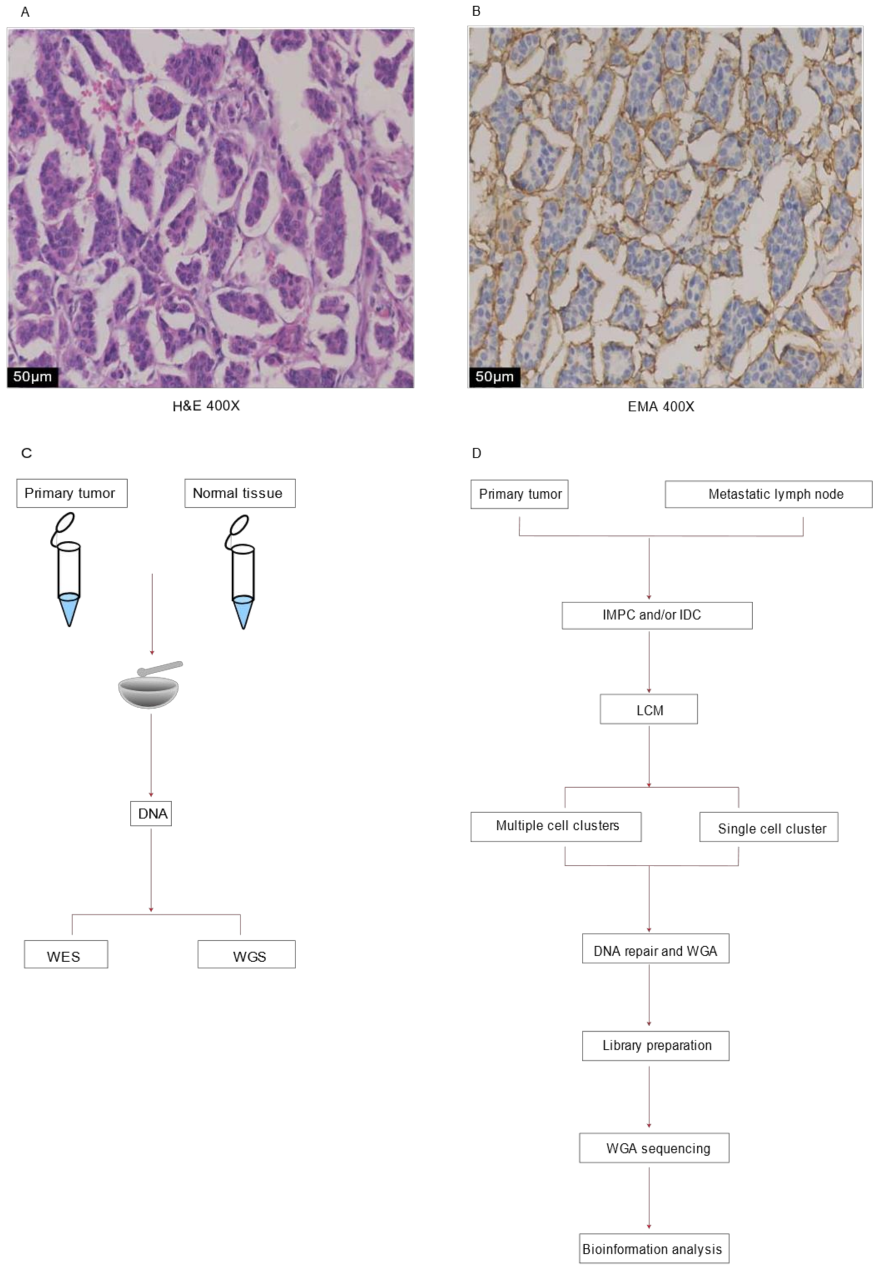

Supplementary Figure 1 The morphological characteristics of IMPC tumor cells and schematic showing the workflow of the sample collection and sequencing strategy for 17 frozen IMPC and 29 FFPE IMPC samples. A Image of H&E staining in IMPC tumor cells. The tumor cells of IMPC were composed of cell clusters surrounded by empty spaces; x400 magnification. All IMPC samples were confirmed by H&E. B Immunohistochemical (IHC) staining for epithelial membrane antigen (EMA), the cell clusters of IMPC displayed the typical reversed polarity growth pattern, as highlighted by EMA staining; x400 magnification. All IMPC samples were confirmed by EMA. C DNA was collected from 17 IMPC tumor-

normal tissue pairs for WES and WGS. D Twenty-nine FFPE IMPCs, including 8 pure IMPCs and 21 mixed IMPC-IDCs, were used to isolate IMPC and/or IDC cell clusters from primary tumors and matched lymph node metastases by LCM for WGS.

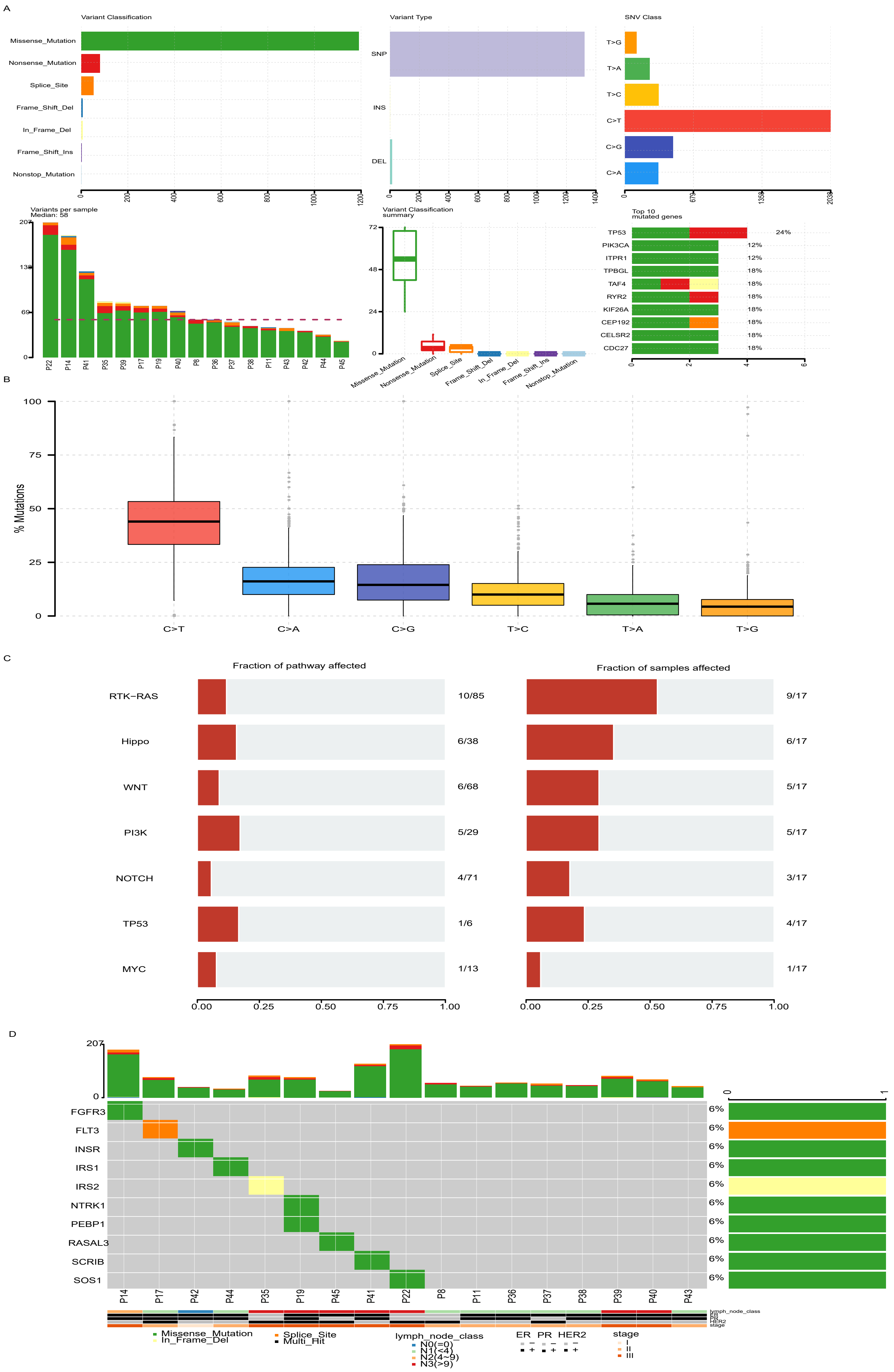
A
Variant Classification
Missense_Mutation
Nonsense_Mutation
Splice_Site
Frame_Shift_Del
In_Frame_Del
Frame_Shift_Ins
Nonstop_Mutation
Variant Type
SNP
INS
DEL
SNV Class
T>G
T>A
T>C
C>T
C>G
C>A
Variants per sample
Median: 58
Variant Classification summary
Top 10 mutated genes
TP53 24%
PIK3CA 12%
ITPR1 12%
TPBGL 18%
TAF4 18%
RYR2 18%
KIF26A 18%
CEP192 18%
CELSR2 18%
CDC27 18%
B
% Mutations
C>T
C>A
C>G
T>C
T>A
T>G
C
Fraction of pathway affected
Fraction of samples affected
RTK-RAS 10/85 9/17
Hippo 6/38 6/17
WNT 6/68 5/17
PI3K 5/29 5/17
NOTCH 4/71 3/17
TP53 1/6 4/17
MYC 1/13 1/17
D
FGFR3 6%
FLT3 6%
INSR 6%
IRS1 6%
IRS2 6%
NTRK1 6%
PEBP1 6%
RASAL3 6%
SCRIB 6%
SOS1 6%
P14 P17 P42 P44 P35 P19 P45 P41 P22 P8 P11 P36 P37 P38 P39 P40 P43
lymph_node_class
ER
PR
HER2
stage
Missense_Mutation
In_Frame_Del
Splice_Site
Multi_Hit
N0(=0)
N1(<4)
N2(4~9)
N3(>9)
I
II
III

Supplementary Figure 2 Somatic genetic alteration profile of IMPC and TCGA breast cancer tissues. (related to Fig. 1) A Genomic somatic mutation in 17 freshly frozen IMPC tumor-normal tissue pairs, including variant classification, variant type, SNV class of 6 categories (C > A, C > G, C > T, A > C, A > G, and A > T) according to the principle of base complementary pairing, variants per sample, variant classification and top 10 mutated genes (with the box plot center, box, whiskers, and points corresponding to the median, interquartile range, 1.5× interquartile range, and outliers, respectively). B The distribution of mutational signatures in TCGA breast cancer (978 samples) (with the box plot center, box, whiskers, and points corresponding to the median, interquartile range, 1.5× interquartile range, and outliers, respectively). C Analysis of the enrichment of non-silent somatic mutations detected in IMPC bulk frozen samples in oncogenic signaling pathways. D RTK-RAS pathway was enriched in most samples (9/17). The 10 genes (Y-axis) represent the number of mutated genes involved in the RTK-RAS pathway in the IMPC sample.

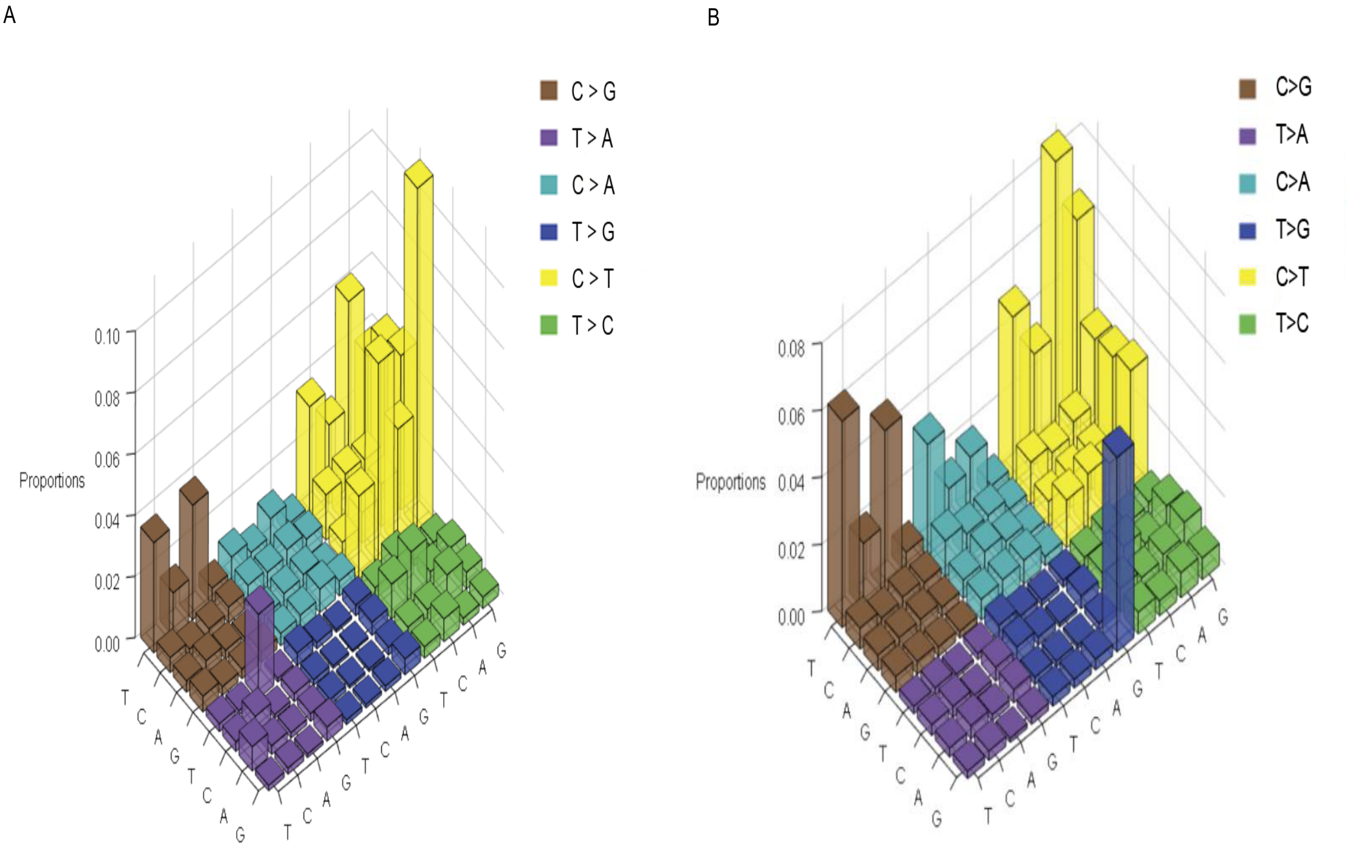

Supplementary Figure 3 The analysis of 96 mutational signatures based on the three-nucleotide context in IMPC and TCGA breast cancer tissues. A The signatures of IMPC. B The signatures of TCGA breast cancer. Six categories of mutations (C > G, T> A, C > A, T > G, C > T, and T > C) are marked with different colors. The horizontal axis indicates different bases, and the vertical axis indicates the proportions of mutational categories.

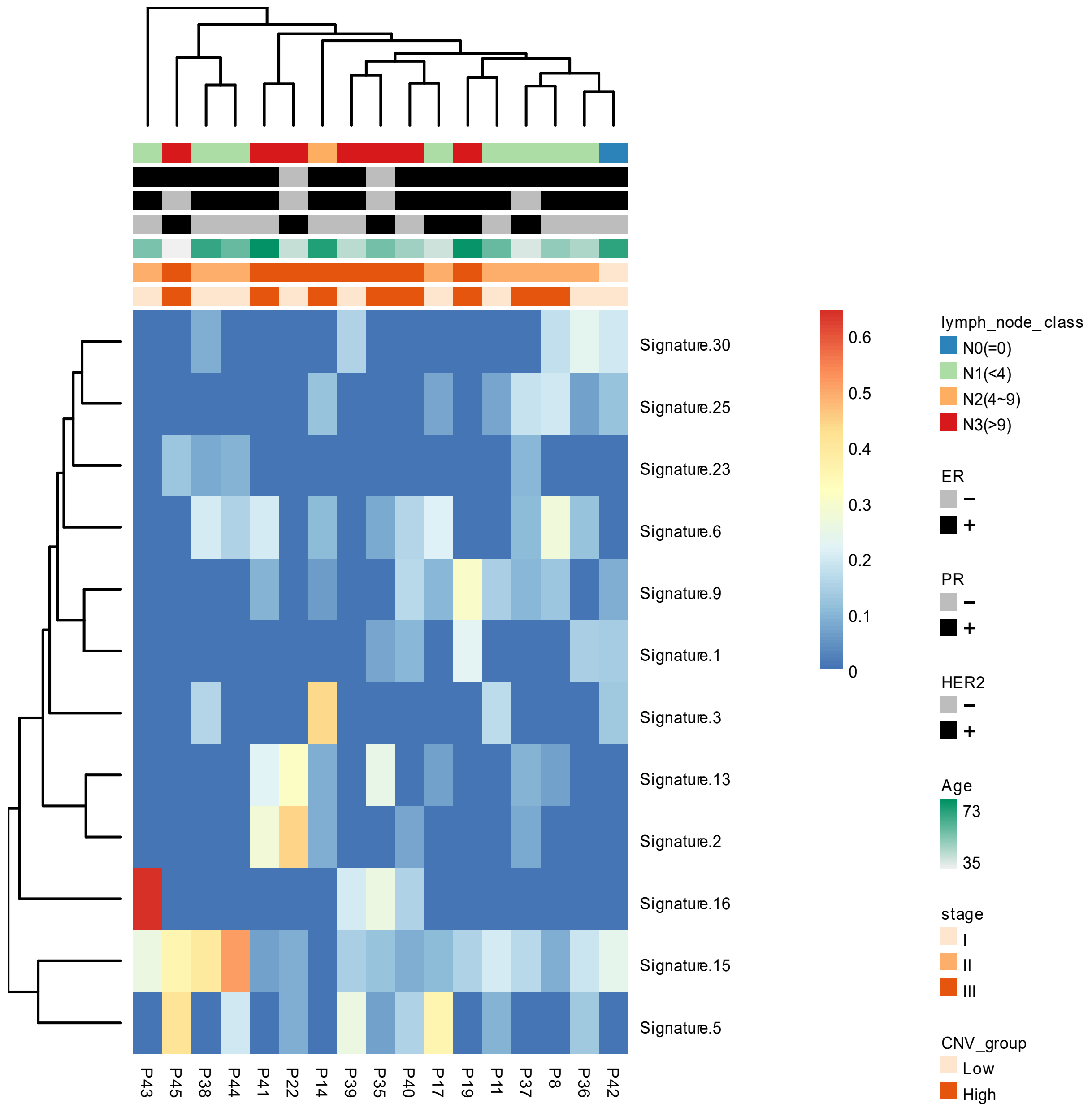

Supplementary Figure 4 The mutational signatures in 17 IMPC samples analyzed using deconstructSigs. Twelve mutational signatures were identified. The horizontal axis indicates different samples, and the vertical axis indicates different signatures. Notably, 0-0.6 represents the proportion of the distribution of mutational signatures in different samples.

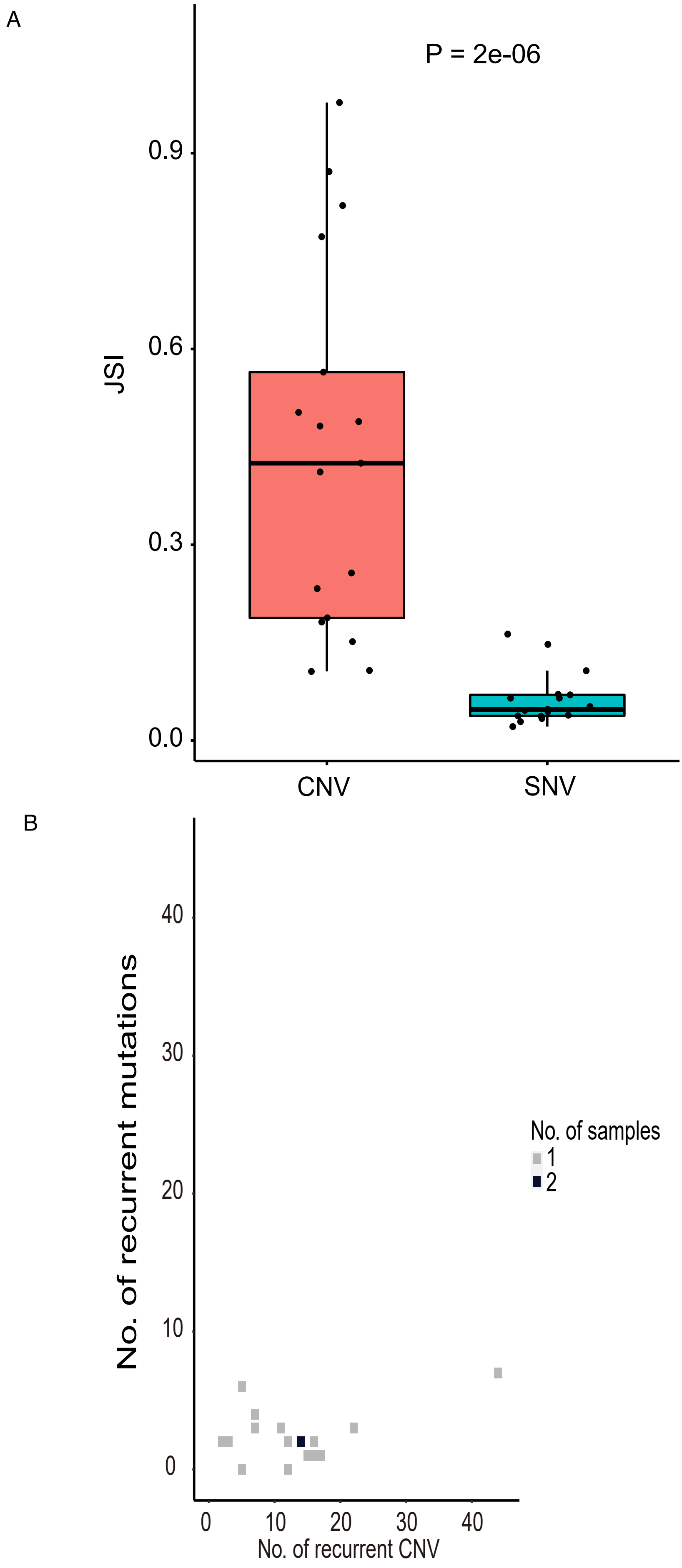

Supplementary Figure 5 The association between CNVs and SNVs in IMPC (related to Fig. 2). A JSI between CNVs and SNVs (n=17). The JSI value represents the mutational similarity of the tumor metastatic

potential between CNVs and SNVs. The JSI values of CNVs are significantly higher than the JSI values of SNVs. Wilcoxon Rank Sum and Signed Rank Tests, $P<0.0001$, (with the box plot center, box, whiskers, and points corresponding to the median, interquartile range,1.5× interquartile range, and outliers, respectively). B Distribution of mutations (SNVs) and CNVs in the IMPC dataset. The 17 IMPC samples vary in the number of recurrent CNVs (X-axis) and number of recurrent SNVs (Y-axis). Gray represent one sample. Black represents two samples.

P14

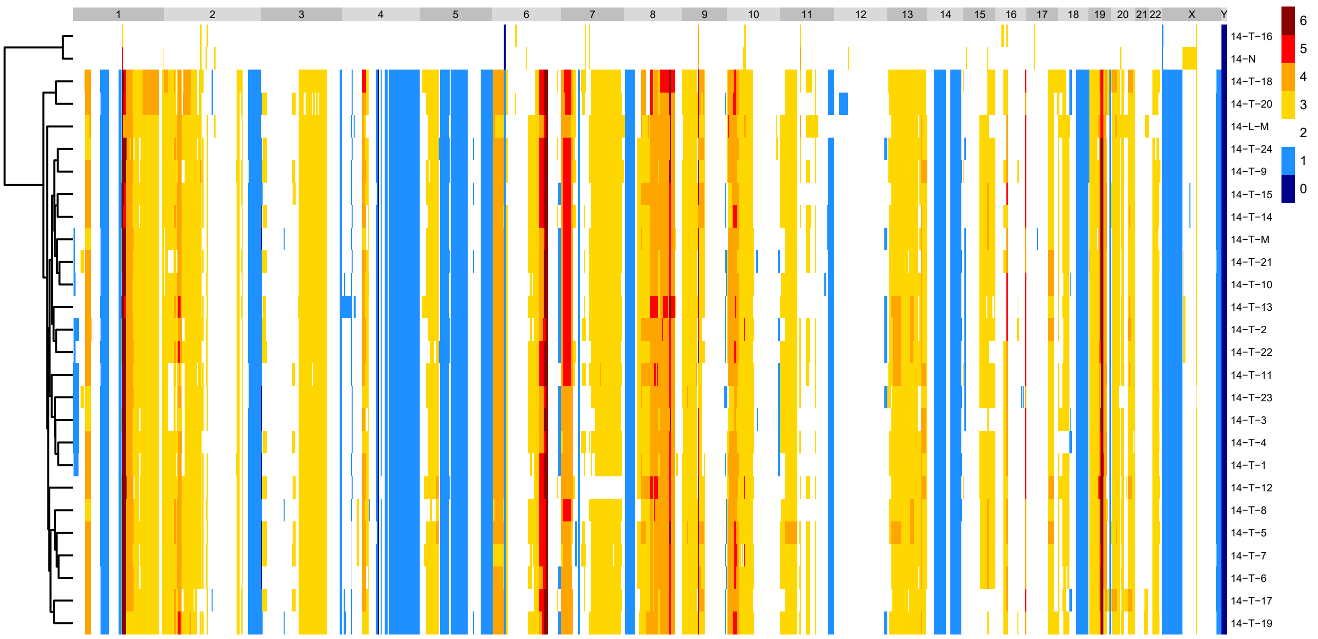
1 2 3 4 5 6 7 8 9 10 11 12 13 14 15 16 17 18 19 20 21 22 X Y
14−T−16
14−N
14−T−18
14−T−20
14−L−M
14−T−24
14−T−9
14−T−15
14−T−14
14−T−M
14−T−21
14−T−10
14−T−13
14−T−2
14−T−22
14−T−11
14−T−23
14−T−3
14−T−4
14−T−1
14−T−12
14−T−8
14−T−5
14−T−7
14−T−6
14−T−17
14−T−19
6 5 4 3 2 1 0

P16

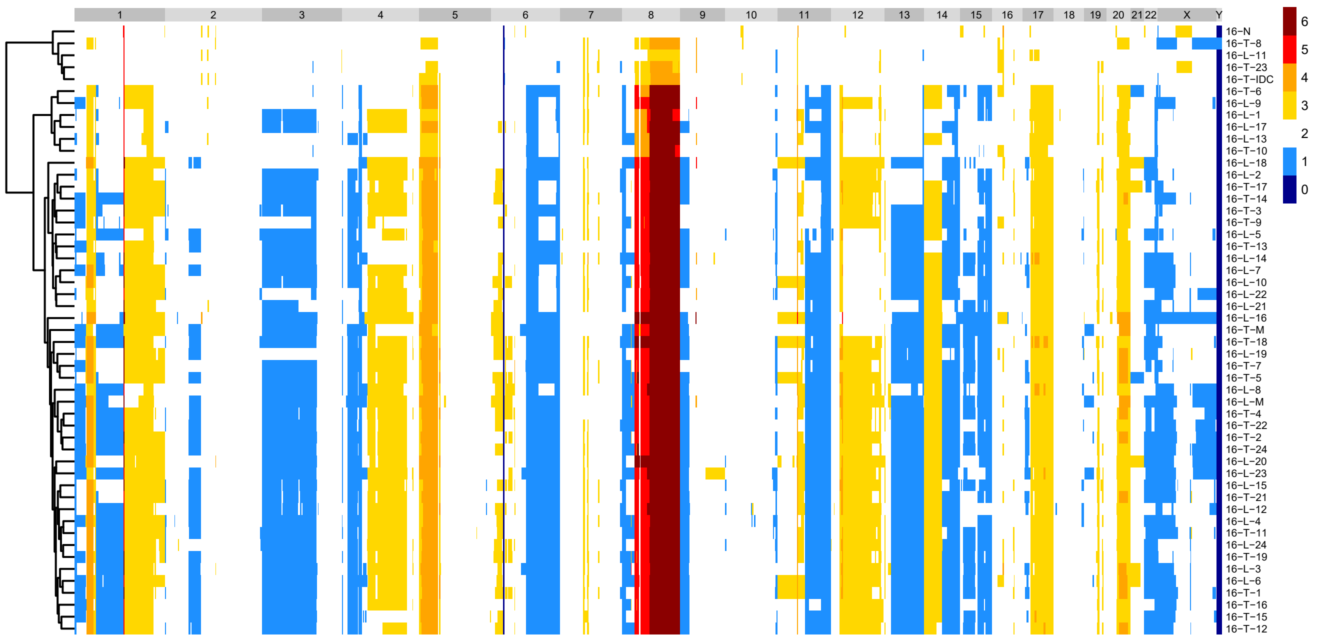
1 2 3 4 5 6 7 8 9 10 11 12 13 14 15 16 17 18 19 20 21 22 X Y
16−N
16−T−8
16−L−11
16−T−23
16−T−IDC
16−T−6
16−L−9
16−L−1
16−L−17
16−L−13
16−T−10
16−L−18
16−L−2
16−T−17
16−T−14
16−T−3
16−T−9
16−L−5
16−T−13
16−L−14
16−L−7
16−L−10
16−L−22
16−L−21
16−L−16
16−T−M
16−T−18
16−L−19
16−T−7
16−T−5
16−L−8
16−L−M
16−T−4
16−T−22
16−T−2
16−T−24
16−L−20
16−L−23
16−L−15
16−T−21
16−L−12
16−L−4
16−T−11
16−L−24
16−T−19
16−L−3
16−L−6
16−T−1
16−T−16
16−T−15
16−T−12
6 5 4 3 2 1 0

P18

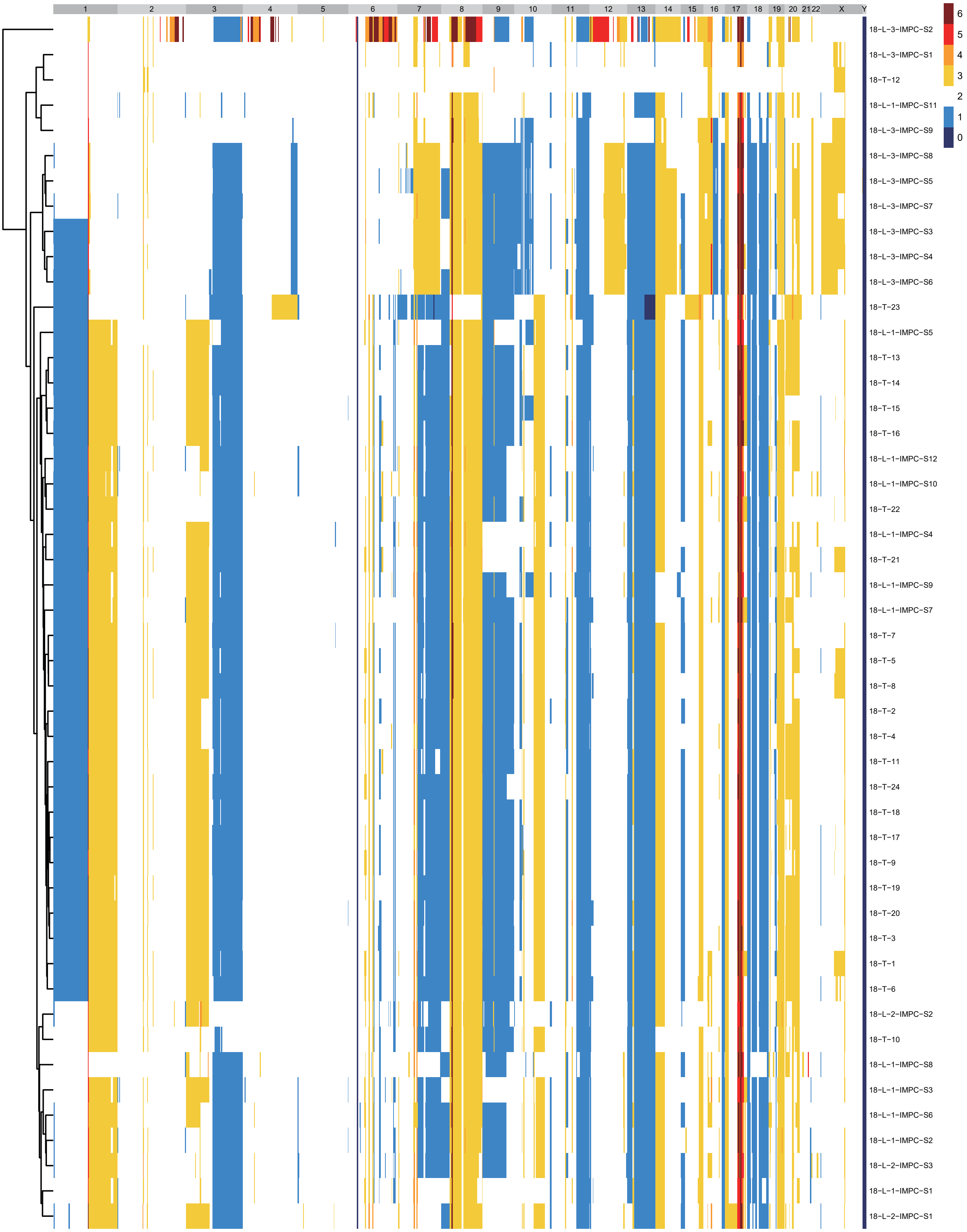
1 2 3 4 5 6 7 8 9 10 11 12 13 14 15 16 17 18 19 20 21 22 X Y
6
5
4
3
2
1
0
18-L-3-IMPC-S2
18-L-3-IMPC-S1
18-T-12
18-L-1-IMPC-S11
18-L-3-IMPC-S9
18-L-3-IMPC-S8
18-L-3-IMPC-S5
18-L-3-IMPC-S7
18-L-3-IMPC-S3
18-L-3-IMPC-S4
18-L-3-IMPC-S6
18-T-23
18-L-1-IMPC-S5
18-T-13
18-T-14
18-T-15
18-T-16
18-L-1-IMPC-S12
18-L-1-IMPC-S10
18-T-22
18-L-1-IMPC-S4
18-T-21
18-L-1-IMPC-S9
18-L-1-IMPC-S7
18-T-7
18-T-5
18-T-8
18-T-2
18-T-4
18-T-11
18-T-24
18-T-18
18-T-17
18-T-9
18-T-19
18-T-20
18-T-3
18-T-1
18-T-6
18-L-2-IMPC-S2
18-T-10
18-L-1-IMPC-S8
18-L-1-IMPC-S3
18-L-1-IMPC-S6
18-L-1-IMPC-S2
18-L-2-IMPC-S3
18-L-1-IMPC-S1
18-L-2-IMPC-S1

P22

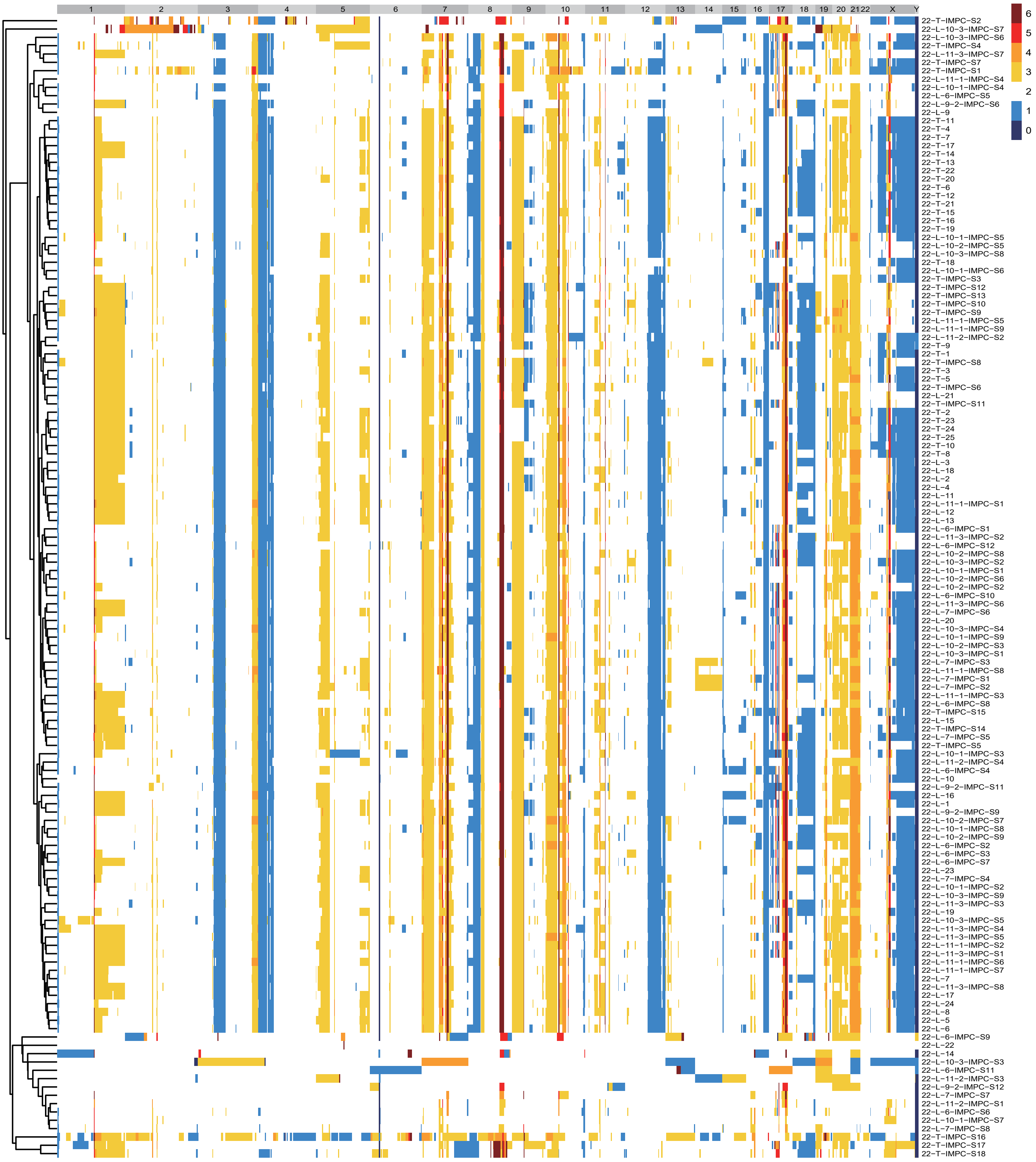
1
2
3
4
5
6
7
8
9
10
11
12
13
14
15
16
17
18
19
20
21
22
X
Y
6
5
4
3
2
1
0
22−T−IMPC−S2
22−L−10−3−IMPC−S7
22−L−10−3−IMPC−S6
22−T−IMPC−S4
22−L−11−3−IMPC−S7
22−T−IMPC−S7
22−T−IMPC−S1
22−L−11−1−IMPC−S4
22−L−10−1−IMPC−S4
22−L−6−IMPC−S5
22−L−9−2−IMPC−S6
22−L−9
22−T−11
22−T−4
22−T−7
22−T−17
22−T−14
22−T−13
22−T−22
22−T−20
22−T−6
22−T−12
22−T−21
22−T−15
22−T−16
22−T−19
22−L−10−1−IMPC−S5
22−L−10−2−IMPC−S5
22−L−10−3−IMPC−S8
22−T−18
22−L−10−1−IMPC−S6
22−T−IMPC−S3
22−T−IMPC−S12
22−T−IMPC−S13
22−T−IMPC−S10
22−T−IMPC−S9
22−L−11−1−IMPC−S5
22−L−11−1−IMPC−S9
22−L−11−2−IMPC−S2
22−T−9
22−T−1
22−T−IMPC−S8
22−T−3
22−T−5
22−T−IMPC−S6
22−L−21
22−T−IMPC−S11
22−T−2
22−T−23
22−T−24
22−T−25
22−T−10
22−T−8
22−L−3
22−L−18
22−L−2
22−L−4
22−L−11
22−L−11−1−IMPC−S1
22−L−12
22−L−13
22−L−6−IMPC−S1
22−L−11−3−IMPC−S2
22−L−6−IMPC−S12
22−L−10−2−IMPC−S8
22−L−10−3−IMPC−S2
22−L−10−1−IMPC−S1
22−L−10−2−IMPC−S6
22−L−10−2−IMPC−S2
22−L−6−IMPC−S10
22−L−11−3−IMPC−S6
22−L−7−IMPC−S6
22−L−20
22−L−10−3−IMPC−S4
22−L−10−1−IMPC−S9
22−L−10−2−IMPC−S3
22−L−10−3−IMPC−S1
22−L−7−IMPC−S3
22−L−11−1−IMPC−S8
22−L−7−IMPC−S1
22−L−7−IMPC−S2
22−L−11−1−IMPC−S3
22−L−6−IMPC−S8
22−T−IMPC−S15
22−L−15
22−T−IMPC−S14
22−L−7−IMPC−S5
22−T−IMPC−S5
22−L−10−1−IMPC−S3
22−L−11−2−IMPC−S4
22−L−6−IMPC−S4
22−L−10
22−L−9−2−IMPC−S11
22−L−16
22−L−1
22−L−9−2−IMPC−S9
22−L−10−2−IMPC−S7
22−L−10−1−IMPC−S8
22−L−10−2−IMPC−S9
22−L−6−IMPC−S2
22−L−6−IMPC−S3
22−L−6−IMPC−S7
22−L−23
22−L−7−IMPC−S4
22−L−10−1−IMPC−S2
22−L−10−3−IMPC−S9
22−L−11−3−IMPC−S3
22−L−19
22−L−10−3−IMPC−S5
22−L−11−3−IMPC−S4
22−L−11−3−IMPC−S5
22−L−11−1−IMPC−S2
22−L−11−3−IMPC−S1
22−L−11−1−IMPC−S6
22−L−11−1−IMPC−S7
22−L−7
22−L−11−3−IMPC−S8
22−L−17
22−L−24
22−L−8
22−L−5
22−L−6
22−L−6−IMPC−S9
22−L−22
22−L−14
22−L−10−3−IMPC−S3
22−L−6−IMPC−S11
22−L−11−2−IMPC−S3
22−L−9−2−IMPC−S12
22−L−7−IMPC−S7
22−L−11−2−IMPC−S1
22−L−6−IMPC−S6
22−L−10−1−IMPC−S7
22−L−7−IMPC−S8
22−T−IMPC−S16
22−T−IMPC−S17
22−T−IMPC−S18

P23

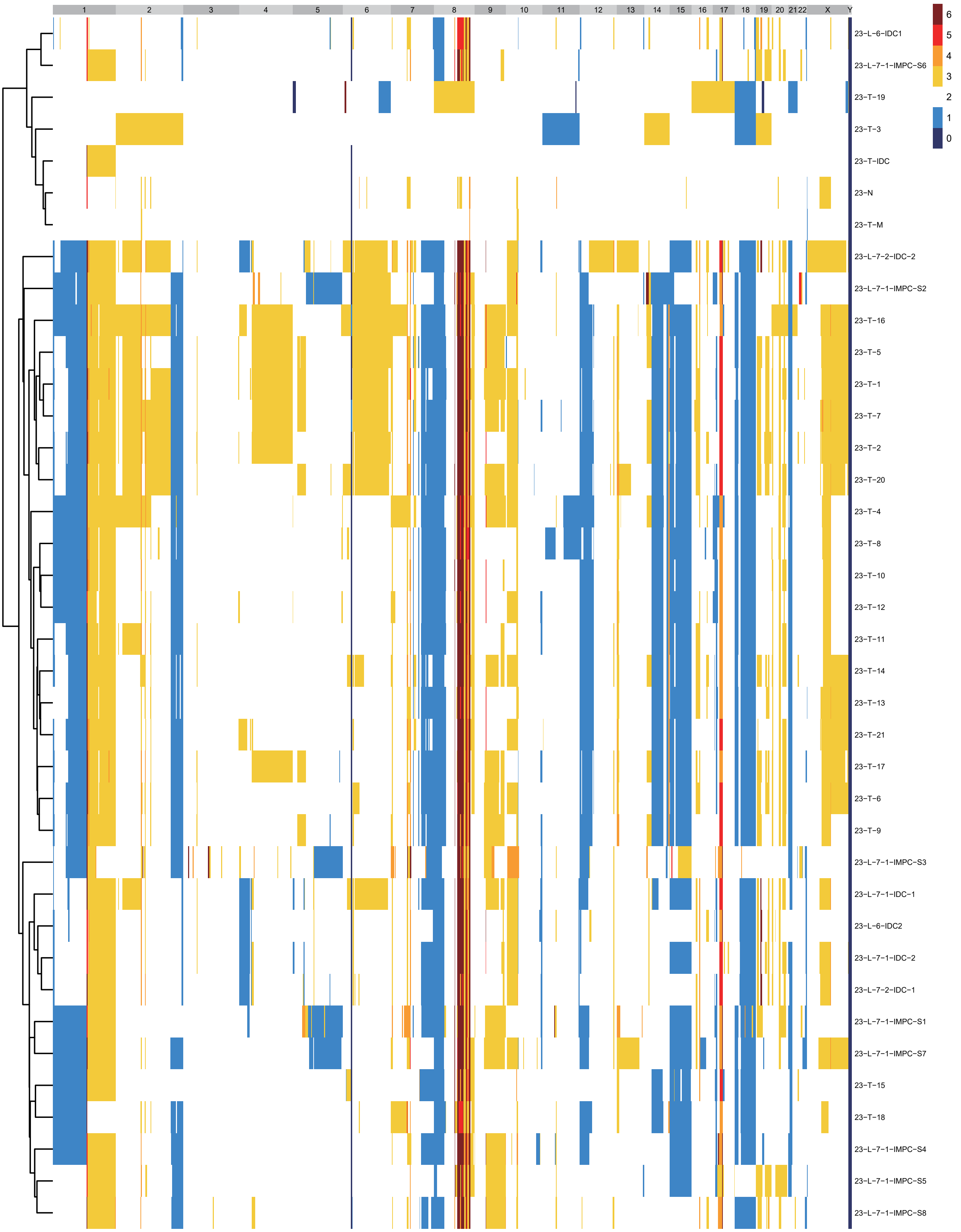

Supplementary Figure 6 Heatmap of copy number profiles (related to Fig. 5). Unsupervised clustering of patients P14, P16, P18, P22, and P23 based on CNV similarity between different cell clusters, indicating the different evolutionary stages from which the cells were isolated.

A

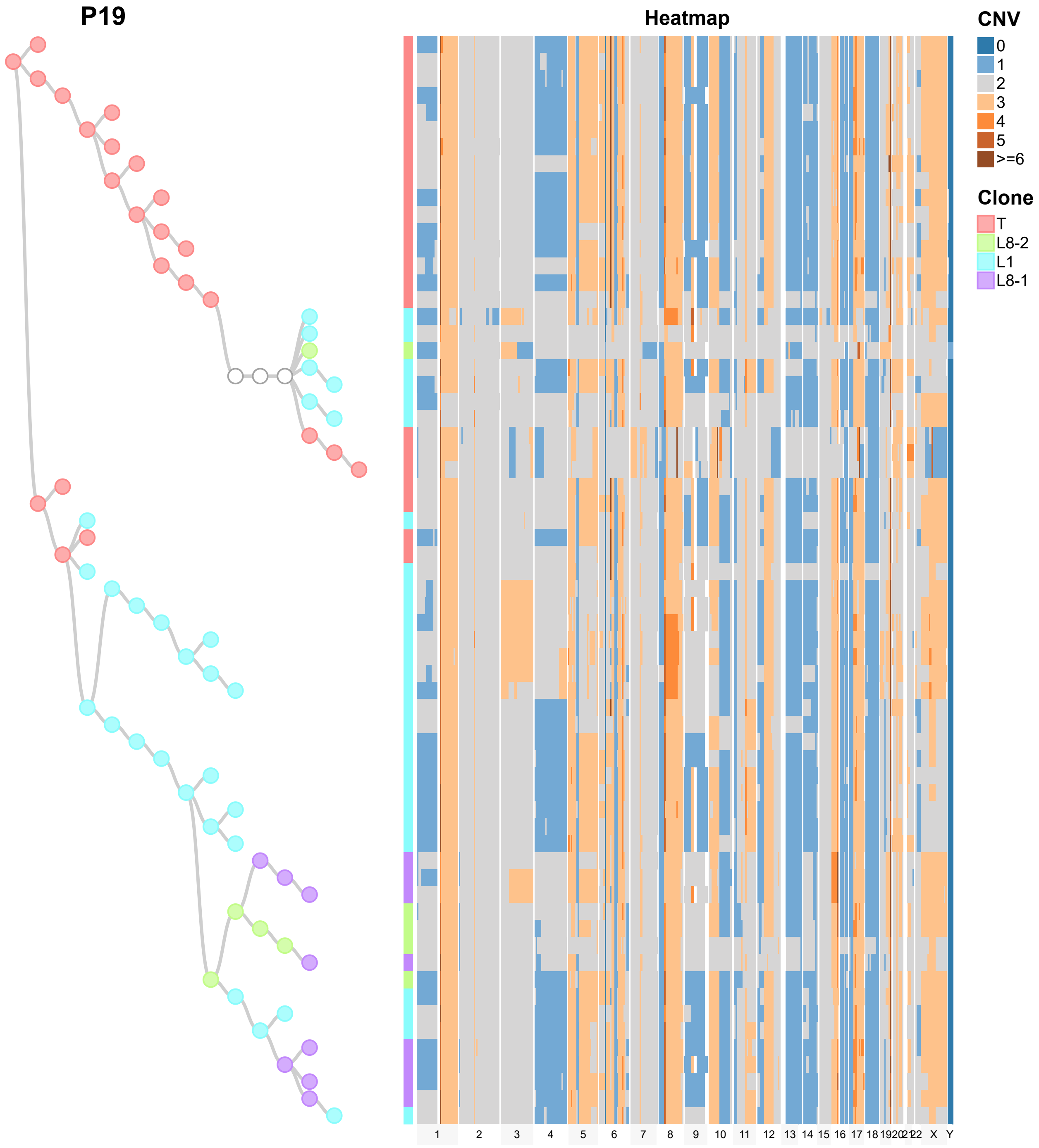
P19
Heatmap
CNV
0
1
2
3
4
5
>=6
Clone
T
L8-2
L1
L8-1
1 2 3 4 5 6 7 8 9 10 11 12 13 14 15 16 17 18 19 20 21 22 X Y

B

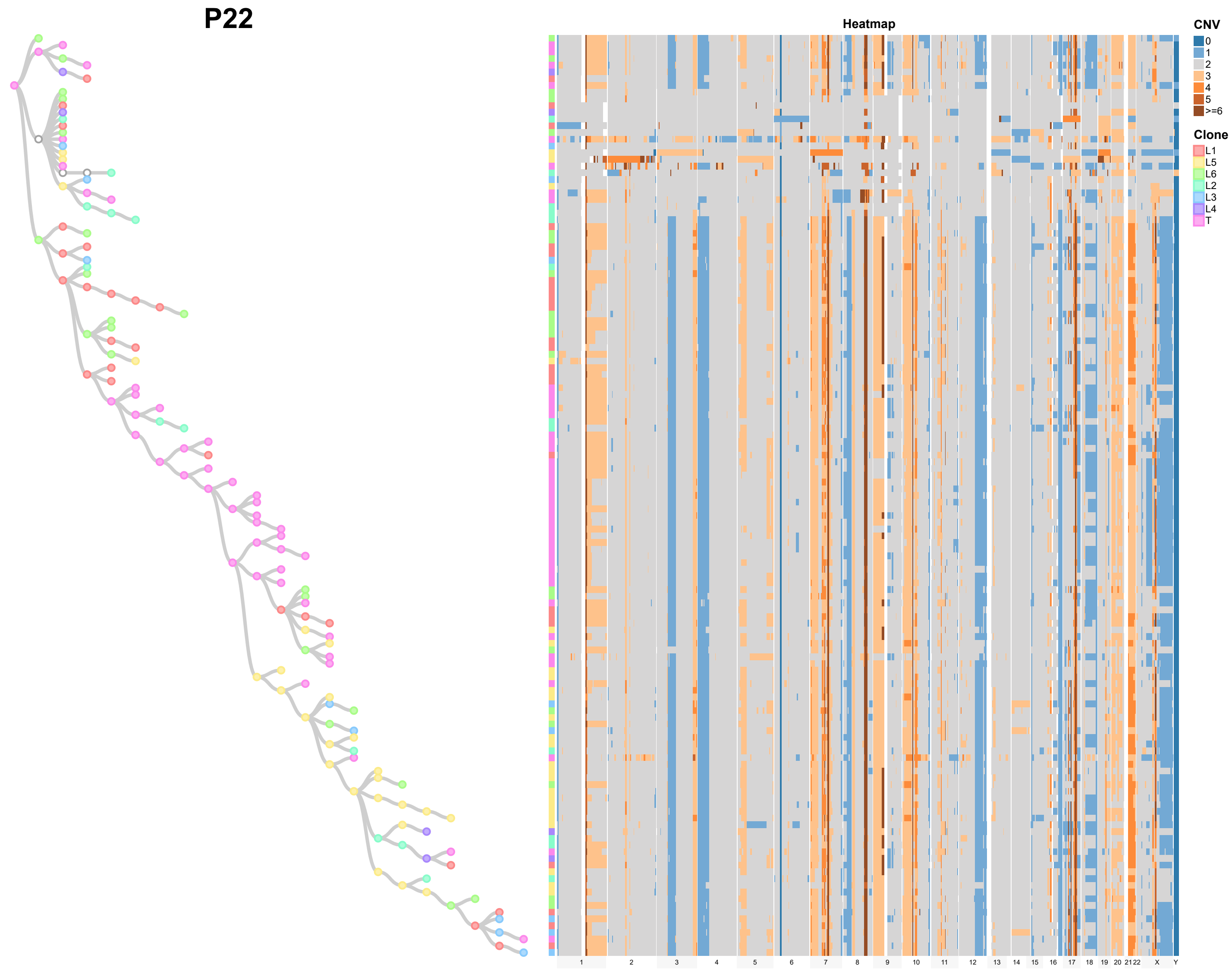
P22
Heatmap
CNV
0
1
2
3
4
5
>=6
Clone
L1
L5
L6
L2
L3
L4
T
1 2 3 4 5 6 7 8 9 10 11 12 13 14 15 16 17 18 19 20 21 22 X Y

C

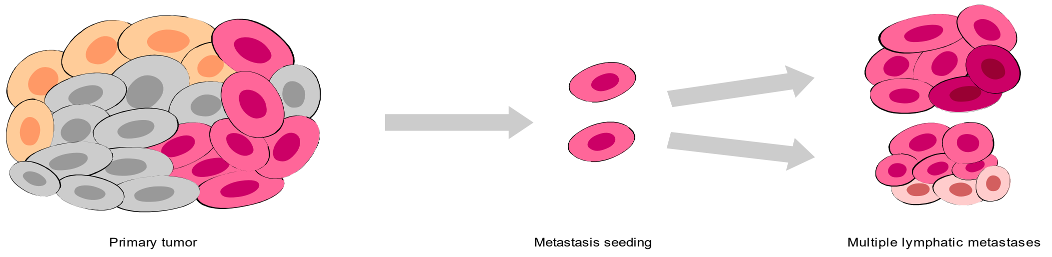
Primary tumor
Metastasis seeding
Multiple lymphatic metastases

Supplementary Figure 7 IMPC cell cluster phylogenetic tree for P19 and P22 (related to Fig. 6). A-B The heatmap depicts genome-wide CNVs (columns) across cell clusters (rows). Copy number gains and losses are indicated by brown and blue color gradients, respectively. The tree illustrates the evolutionary relationship based on the minimum spanning tree method. Colored annotations indicate the region membership of cell clusters. C The metastatic path of an IMPC cell cluster from the primary tumor to multiple lymph node metastases. Colors represent distinct clones in the primary tumor. The red clone is the metastatic seed, which may continue to evolve when it migrates to the lymph nodes.

P19 L1

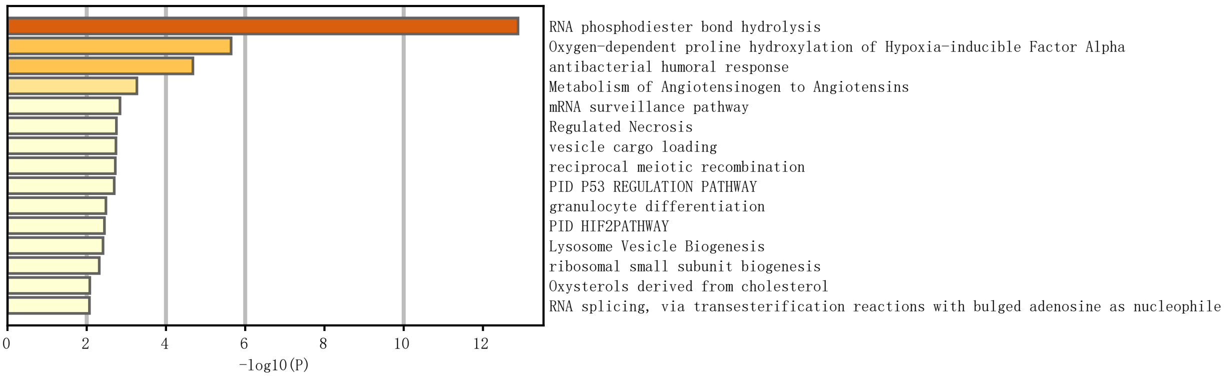

P19 L2

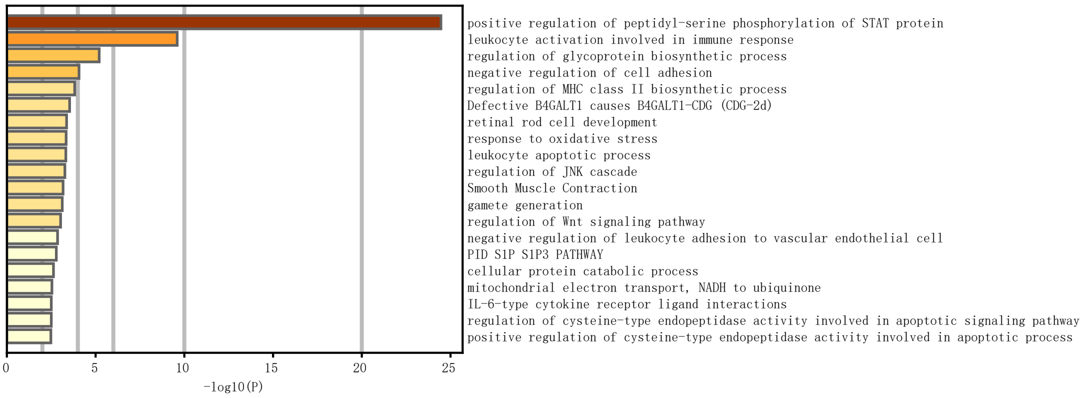

P19 L3

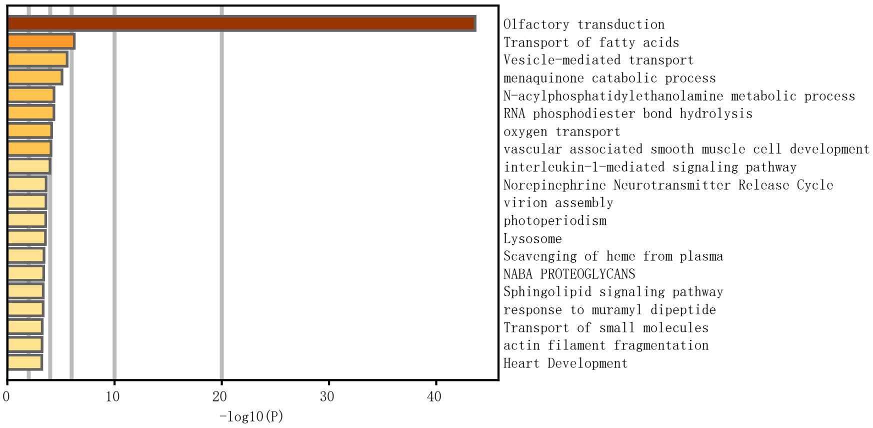

P19 L4

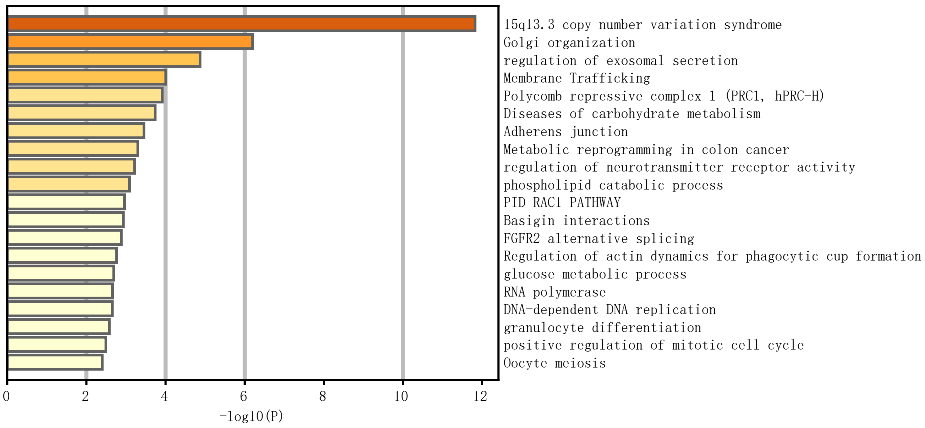

P19 L5

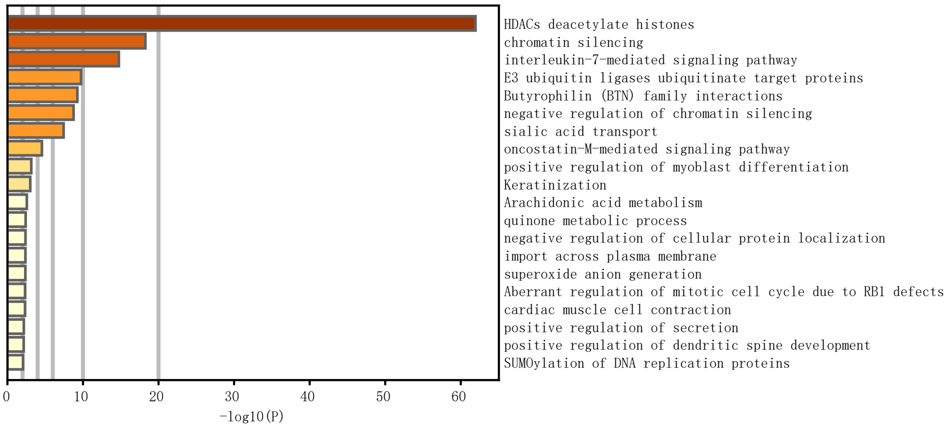

P19 L6

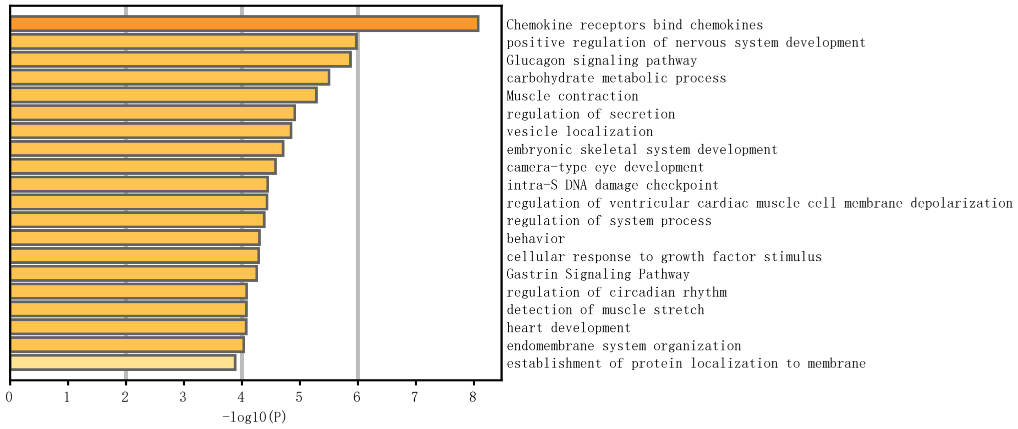

P22 L1

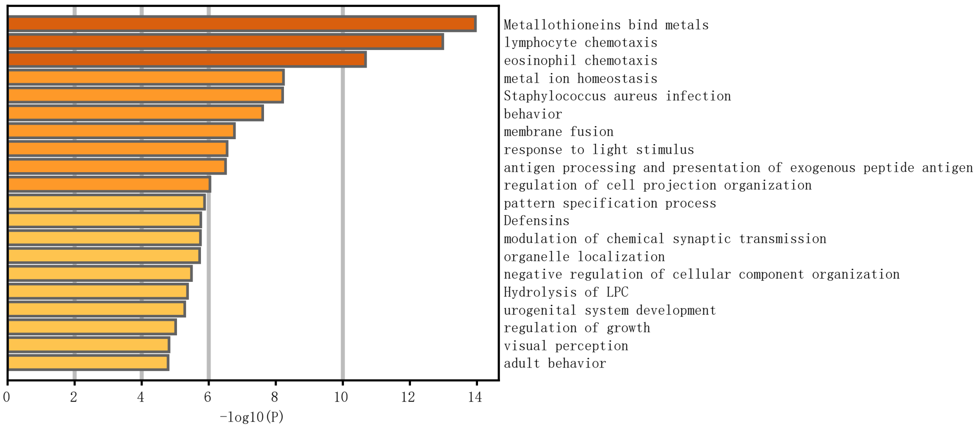

P22 L2

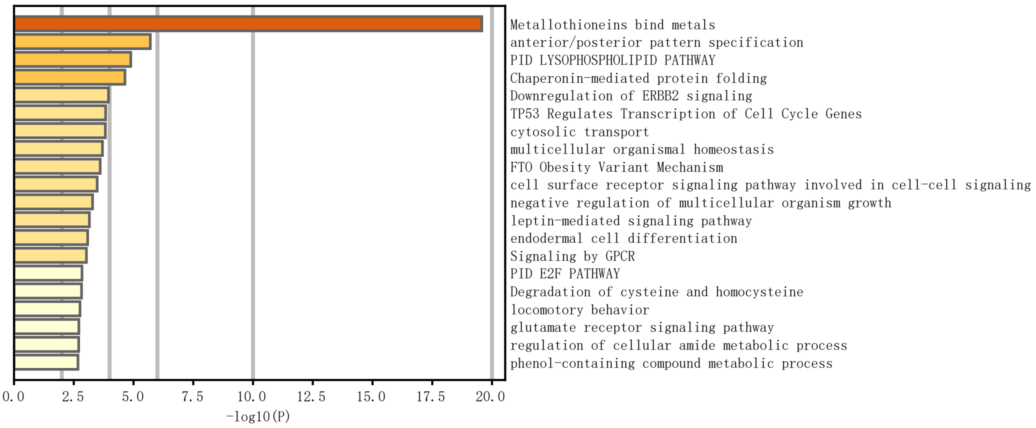

P22 L3

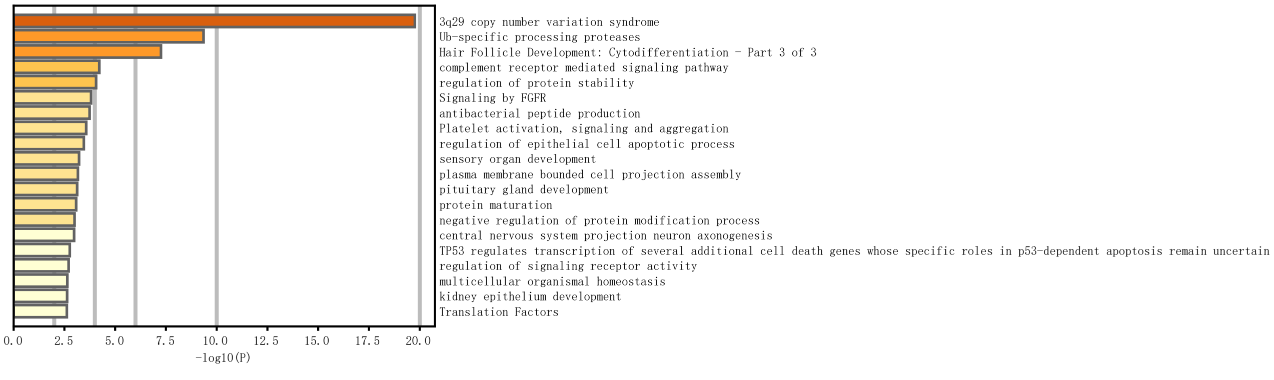

P22 L4

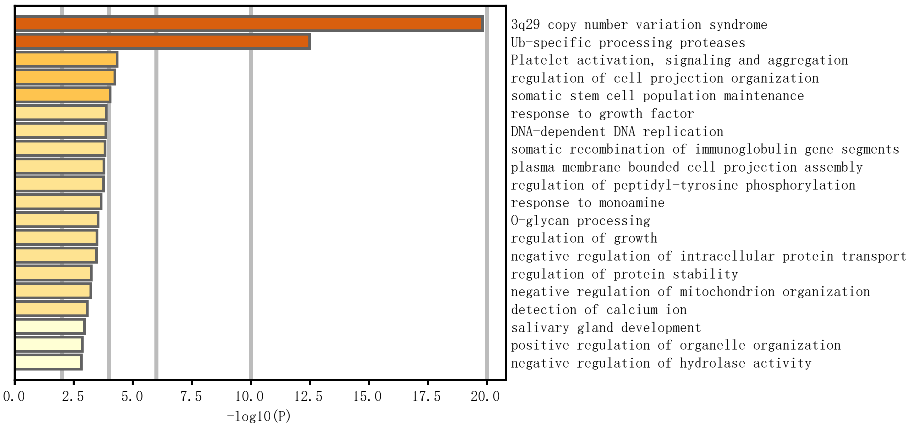

Supplementary Figure 8 Enrichment within multiple lymph node metastatic clones (related to Fig. 6). Bar graph showing the functional enrichment of specific CNVs in genes in multiple lymph node metastatic. The horizontal axis indicates the significance of enrichment; the vertical axis indicates the enriched pathway. The clones were determined from the development tree present in **Supplementary Fig. 5**. Each clone may be distributed between different lymph node metastatic lesions. The well-adopted hypergeometric test and Benjamini-Hochberg p-value correction algorithm to identify all ontology terms that contain a significantly greater number of genes in common with an input list than expected by chance, two-sided.

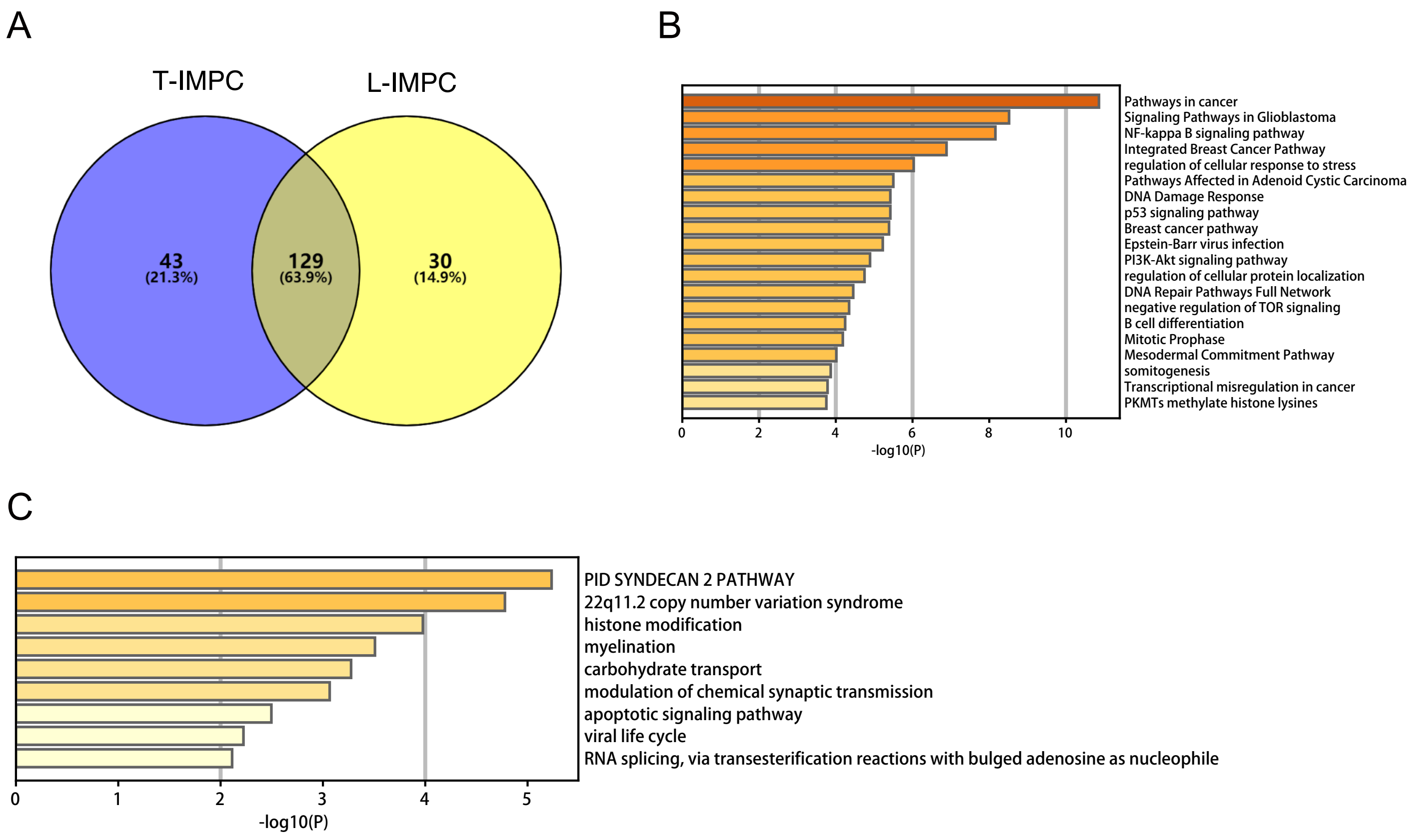

Supplementary Figure 9 Evolutionary relationship between multiple lymph node metastases (related to Fig. 6). A Venn diagram showing the common and unique genes with CNVs between the primary IMPC and lymph node metastases from P18. The percentage of genes included in each category is labeled. B-C Functional enrichment of two lymph node metastatic clones, one clone including L2 and L3 and the other clone including L1. The enriched pathway revealed that gene function is related to lymph node activation in early metastases (L2 and L3, B); in late metastases (L1, C), gene function is related to tumor colonization and immune disorders. The well-adopted hypergeometric test and Benjamini-Hochberg p-value correction algorithm to identify all ontology terms that contain a significantly greater number of genes in common with an input list than expected by chance, two-sided.

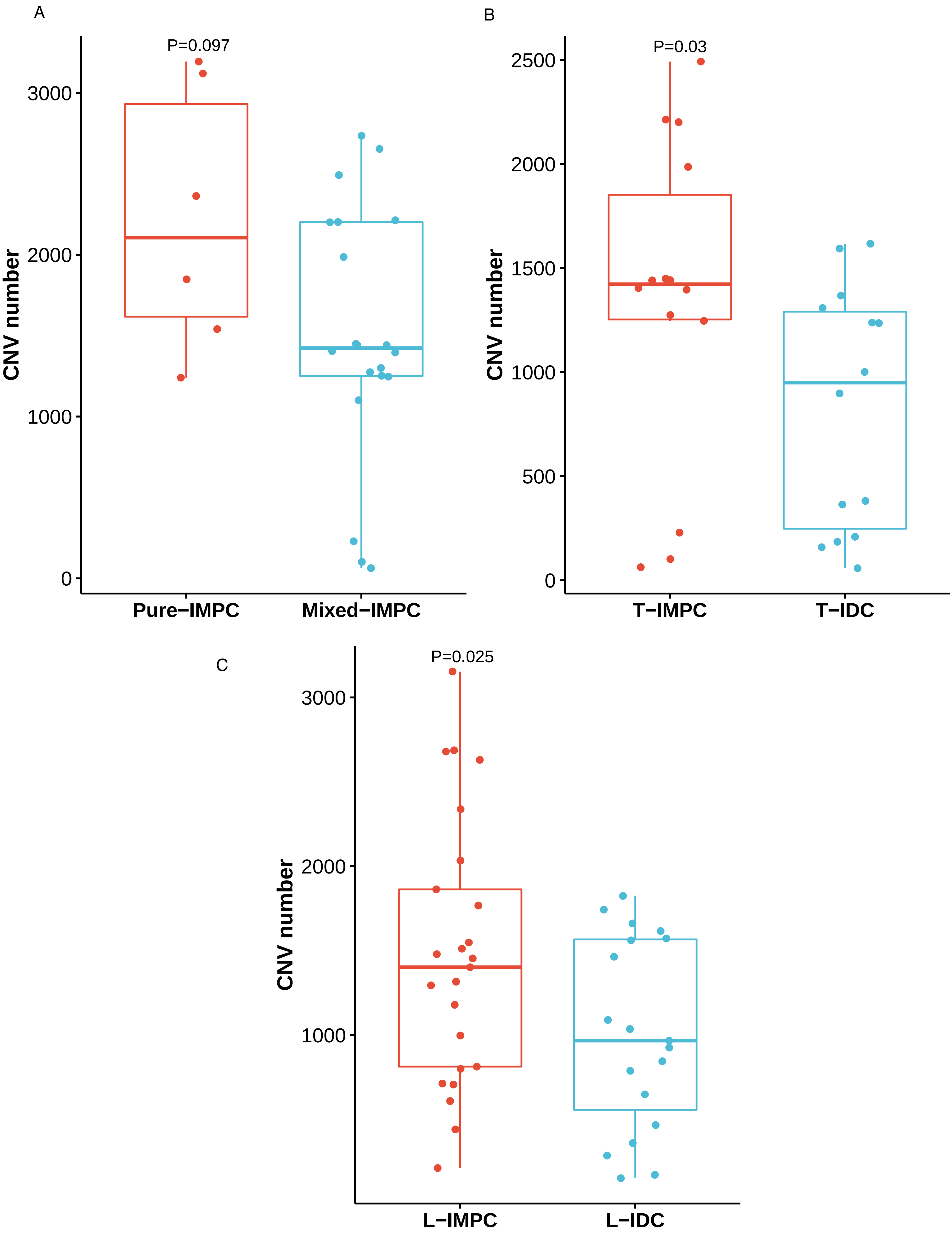

Supplementary Figure 10 CNV number between different component of IDC and IMPC. Box plot of CNV number between A. Pure and mixed IMPC, B. Primary tumor IMPC and IDC, C. Lymph node IMPC and IDC. Student's *t*-test, pure VS. mixed, P=0.097, primary tumor, P=0.03, lymph node, P = 0.025

Supplementary Table 1 The difference between CNV groups and the lymph node metastatic stage.

Groups	Patient ID	Metastatic LN number	Total LN number	LN stage	P-value (Mann-Whitney U-test)
CNV high group	P45	26	30	N3	0.045
	P8	3	26	N1	
	P19	16	21	N3	
	P41	12	17	N3	
	P37	2	10	N1	
	P40	16	22	N3	
	P35	15	18	N3	
	P14	6	15	N2	
CNV low group	P11	3	11	N1	
	P36	3	19	N1	
	P17	1	23	N1	
	P43	2	21	N1	
	P42	0	15	N0	
	P22	16	21	N3	
	P38	2	24	N1	
	P44	2	15	N1	
	P39	18	21	N3	

The CNV high group includes 8 samples, and N3 accounts for 5/8 samples; the CNV low group includes 9 samples, but N3 only accounts for 2/9 samples. The P-value was calculated using the Mann-Whitney U-test, two-sided. $P < 0.05$ was considered to indicate statistical significance. LN: lymph node.

Supplementary Table 2 Correlation between the expression of IGSF9, PRDM16 and ALDH2 proteins and IMPC lymph node metastatic stage.

	LN stage				R	P value
	N0	N1	N2	N3		
IGSF9					-0.32	< 0.01
High	14	14	8	10		
Low	6	7	9	18		
PRDM16					-0.336	< 0.01
High	12	2	2	4		
Low	8	19	15	24		
ALDH2					0.262	< 0.05
High	4	13	11	17		
Low	16	8	6	11		

P-values were calculated for the Spearman correlation coefficient, two-sided. The exact P values of IGSF9, PRDM16 and ALDH2 proteins are 0.003, 0.002 and 0.015, respectively. $P < 0.05$ was considered to indicate statistical significance. LN: lymph node.

Supplementary Table 3 A complete list of custom-designed primers, including numbers and sequences.

List	PCR product size(bp)	Forward	Reverse
1	100	GTTCCAATATGATTCCACCC	CTCCTGGAAGATGGTGATGG
2	200	AGGTGGAGCGAGGCTAGC	TTTTGCGGTGGAAATGTCCT
3	300	AGGTGAGACATTCTTGCTGG	TCCACTAACCAGTCAGCGTC
4	400	ACAGTCCATGCCATCACTGC	GCTTGACAAAGTGGTCGTTG

There are four primers; each primer included a forward primer and a reverse primer. The primers were randomly selected from the genome to assess the DNA integrity of FFPE samples. The PCR products 300-bp or 400-bp were deemed to be good quality and were selected for this study.
