# Supplementary material for: Genomic alterations and evolution of cell clusters in metastatic invasive micropapillary carcinoma of the breast
Source: Nat Commun. 2022 Jan 10;13:111. doi: 10.1038/s41467-021-27794-4 (PMC8748639; doi:10.1038/s41467-021-27794-4)
Supplement: Supplementary file 3 — Description of Additional Supplementary Files [file 41467_2021_27794_MOESM3_ESM.pdf]

Description of Additional Supplementary Files

Supplementary Data 1: Clinicopathologic characteristics and sequencing information of freshly frozen tissues of 17 IMPC patients.

Supplementary Data 2: Non-silent somatic mutations in IMPC.

Supplementary Data 3: The mutational signatures in 17 IMPC samples.

Supplementary Data 4: The genomic copy number variations in 17 IMPC samples.

Supplementary Data 5: Clinicopathological characteristics and sequencing information of formalin-fixed paraffin-embedded (FFPE) tissues in 29 IMPC patients.

Supplementary Data 6: The copy number variations between pure IMPC and mixed IMPC-IDC; the copy number variations between primary IMPC and primary IDC in mixed IMPC-IDC; the copy number variations between metastatic-IMPC and metastatic-IDC in mixed IMPC-IDC.

Supplementary Data 7: The copy number variations in patient P23, which has both IMPC and IDC components in the primary tumor and lymph node metastases.

Supplementary Data 8: IGSF9, PRDM16 and ALDH2 protein expression versus the clinicopathological parameters of 86 IMPC patients.

Supplementary Data 9: Univariate and multivariate Cox regression models were applied to analyze the predictors for overall survival (OS) and disease-free survival (DFS) of 86 IMPC patients.
