# Supplementary material for: Genomic alterations and evolution of cell clusters in metastatic invasive micropapillary carcinoma of the breast
Source: Nat Commun. 2022 Jan 10;13:111. doi: 10.1038/s41467-021-27794-4 (PMC8748639; doi:10.1038/s41467-021-27794-4)
Supplement: Supplementary file 13 — Reporting Summary [file 41467_2021_27794_MOESM13_ESM.pdf]

nature portfolio

Corresponding author(s): Li Fu, Guibo Li

Last updated by author(s): Oct 21, 2021

Reporting Summary

Nature Portfolio wishes to improve the reproducibility of the work that we publish. This form provides structure for consistency and transparency in reporting. For further information on Nature Portfolio policies, see our Editorial Policies and the Editorial Policy Checklist.

Statistics

For all statistical analyses, confirm that the following items are present in the figure legend, table legend, main text, or Methods section.

n/a	Confirmed	
☐	☒	The exact sample size (*n*) for each experimental group/condition, given as a discrete number and unit of measurement
☐	☒	A statement on whether measurements were taken from distinct samples or whether the same sample was measured repeatedly
☐	☒	The statistical test(s) used AND whether they are one- or two-sided<br>*Only common tests should be described solely by name; describe more complex techniques in the Methods section.*
☐	☒	A description of all covariates tested
☐	☒	A description of any assumptions or corrections, such as tests of normality and adjustment for multiple comparisons
☐	☒	A full description of the statistical parameters including central tendency (e.g. means) or other basic estimates (e.g. regression coefficient) AND variation (e.g. standard deviation) or associated estimates of uncertainty (e.g. confidence intervals)
☐	☒	For null hypothesis testing, the test statistic (e.g. *F*, *t*, *r*) with confidence intervals, effect sizes, degrees of freedom and *P* value noted<br>*Give P values as exact values whenever suitable.*
☒	☐	For Bayesian analysis, information on the choice of priors and Markov chain Monte Carlo settings
☒	☐	For hierarchical and complex designs, identification of the appropriate level for tests and full reporting of outcomes
☐	☒	Estimates of effect sizes (e.g. Cohen's *d*, Pearson's *r*), indicating how they were calculated

Our web collection on statistics for biologists contains articles on many of the points above.

Software and code

Policy information about availability of computer code

Data collection	No software was used during data collection
Data analysis	Genome Analysis Toolkit (GATK; v3.4, http://www.broadinstitute.org/gatk); Mutect (v1.1.4); Samtools (v1.9); Edico Genome DRAGEN (v0x01011098); ANNOVAR (v20170716); MutSigCV (v3.1); Control-FREEC(v8.0); Gistic2.0; MEDALT (v1.0); Metascape(http://www.metascape.org); R packages: DNA copy(v1.62.0); survival (v2.30); Tanglegram(v1.5.2); DendExtend(1.15.1); CellScape(1.8.0); TimeScape(1.8.0); Oncoprint of ComplexHeatmap(v2.5.6).

For manuscripts utilizing custom algorithms or software that are central to the research but not yet described in published literature, software must be made available to editors and reviewers. We strongly encourage code deposition in a community repository (e.g. GitHub). See the Nature Portfolio guidelines for submitting code & software for further information.

Data

Policy information about availability of data

All manuscripts must include a data availability statement. This statement should provide the following information, where applicable:

- Accession codes, unique identifiers, or web links for publicly available datasets
- A description of any restrictions on data availability
- For clinical datasets or third party data, please ensure that the statement adheres to our policy

The WES and WGS raw data of frozen tissue and cell clusters have been deposited in European Genome -phenome Archive (EGA) hosted by the EBI and CRG under accession number EGAS00001005231 [https://ega-archive.org/studies/EGAS00001005231], and in CNGB Nucleotide Sequence Archive (CNSA) under accession number: CNP0001660 [https://db.cngb.org/search/project/CNP0001660]. Those human related data are accessed via application to Data Access Committee for

research purpose. Potential users will need to complete and be approved of a data access request and then the raw data can be used according to the terms of the consent, and the data use limitations for the subjects.

Field-specific reporting

Please select the one below that is the best fit for your research. If you are not sure, read the appropriate sections before making your selection.

☒ Life sciences ☐ Behavioural & social sciences ☐ Ecological, evolutionary & environmental sciences

For a reference copy of the document with all sections, see nature.com/documents/nr-reporting-summary-flat.pdf

Life sciences study design

All studies must disclose on these points even when the disclosure is negative.

Sample size	No statistical method or calculation was used to determine the sample size. Our goal was to collect data from a large number of individual cells clusters from several donors across multiple tumor sites. Since we performed cell clusters sequencing from multiple donors, each cell clusters represents a n=1 and we sequenced a total of 442 samples in this study, which we believe is a sufficient sample size for this descriptive study.
Data exclusions	No data were excluded from analysis.
Replication	In this study, we sequenced multiple cell clusters per tissue from multiple donors. By sequencing multiple cell clusters both within and across people, we were able to replicate the patterns described in this manuscript.
Randomization	All cell clusters were sequenced using the same protocol and no treatment was applied to any subset of our cells. Therefore, randomization was not applicable for this study.
Blinding	No treatment or variable was applied to any of our samples as they were all processed equally. Therefore, blinding was not applicable for this study design.

Reporting for specific materials, systems and methods

We require information from authors about some types of materials, experimental systems and methods used in many studies. Here, indicate whether each material, system or method listed is relevant to your study. If you are not sure if a list item applies to your research, read the appropriate section before selecting a response.

Materials & experimental systems

n/a	Involved in the study	
☐	☒	Antibodies
☒	☐	Eukaryotic cell lines
☒	☐	Palaeontology and archaeology
☒	☐	Animals and other organisms
☐	☒	Human research participants
☒	☐	Clinical data
☒	☐	Dual use research of concern

Methods

n/a	Involved in the study	
☒	☐	ChIP-seq
☒	☐	Flow cytometry
☒	☐	MRI-based neuroimaging

Antibodies

Antibodies used	Antibodies were purchased from the following sources: Anti-IGSF9(HPA037753)-Sigma; Anti-PRDM16(DF13303)-Affinity; Anti-ALDH2 (ab108306)-Abcam; Anti-MUC1(EPR1023)(ab109185)-Abcam.
Validation	The IGSF9 (HPA037753, Sigma) antibody has been validated by immunochemistry in human endometrial cancer (https://www.sigmaaldrich.com/catalog/product/sigma/hpa037753?lang=zh&region=CN); the PRDM16 (DF13303, Affinity) antibody has been validated by western blot and immunochemistry (http://www.affbiotech.com/goods-16681-DF13303-PRDM16+Antibody.html); the ALDH2 (ab108306, Abcam) antibody has been validated by immunochemistry human lungcarcinoma (https://www.abcam.cn/aldh2-antibody-epr4493-ab108306.html); the MUC1(ab109185)antibody has been validated in normal human kidney tissues(https://www.abcam.cn/hrp-muc1-antibody-epr1023-ab195936.html).

Human research participants

Policy information about studies involving human research participants

Population characteristics	The human tumor tissues in the study are all collected from breast cancer patients after surgery. The breast cancer tumor

Population characteristics	tissues were selected from the full information database of breast cancer patients at the Department of Breast Cancer Pathology and Research Laboratory, Tianjin Medical University Cancer Hospital, Tianjin, China.
Recruitment	We selected IMPC tumor tissues from the Department of Breast Cancer Pathology and Research Laboratory, Tianjin Medical University Cancer Hospital. All IMPC patients signed the informed consent form and agree to use the tumor tissues for follow-up experimental research.
Ethics oversight	This study has been approved by the Ethics Committee of Tianjin Medical University Cancer Hospital, Tianjin, China.

Note that full information on the approval of the study protocol must also be provided in the manuscript.
